# The Future of Iran with a Decrease in the Total Fertility Rate

**DOI:** 10.34172/aim.33643

**Published:** 2025-04-01

**Authors:** Habibollah Azarbakhsh, Seyed Parsa Dehghani

**Affiliations:** ^1^Department of Epidemiology, Faculty of Health, Ahvaz Jundishapur University of Medical Sciences, Ahvaz, Iran; ^2^School of Medicine, Shiraz University of Medical Sciences, Shiraz, Iran

## Dear Editor,

 Demographic policies in any country are considered the basis of socioeconomic development.^1^ These policies include all demographic trends, morbidity and mortality, reproduction and family formation, distribution, immigration, and, generally, the size and structure of a country’s population.^1^ Fertility rate is one of the factors affecting population changes, and total fertility rate (TFR) is the most important indicator for its estimation, which is defined as the average number of children per woman during her reproductive life.^2^ Iran has experienced one of the fastest declines in fertility. The observed pattern is a pattern in which fertility has decreased in all age groups, in all geographic locations, and in all social groups simultaneously; hence the rate of decline is considered at the national level.^3^

 The global TFR has declined over the past seven decades and has experienced a steady decline since 1950. TFR was 4.97 in 1950, 4.40 in 1970, 3.18 in 1990, 2.72 in 2000, and 2.31 in 2019.^4^ TFR in Iran was 6 in 1974, 5.6 in 1985, 5.2 in 1988, 2.8 in 1995, 2.6 in 1996,^5^ and reached 1.8 in 2011.^6^ Based on the average fertility variable, Iran’s natural growth rate will decrease to almost zero percent in 2045-2050. In addition, it is expected to decrease to less than zero percent from 2050 onwards.^7^

 The decrease in the overall fertility rate in Iran will have many consequences in the coming years, including changes in fertility behaviors and the realization of a small ideal family, increasing the age of marriage in men and women, changes in the age structure of the population, and aging of the population,^8^ shortage of the labor force, an increased disabled population and the needs of this population for care, an increase in non-communicable diseases and the need for diagnostic and treatment facilities due to the aging of the population, the decrease in the resilience of parents and children, reduced consolidation of the family foundation, reduction of family support for the elderly, lack of economic growth and development, moral and educational damages, disturbance in the social communication skills of children of low-population families, mental and psychological problems of the future generation due to not benefiting from the social gifts of kinship (including brothers, sisters, uncles, and aunts), forced migration and cultural disintegration of the destination country and the reduction of the country’s young and elite population.

 Given the decreasing trend of the TFR and the aging of the population in Iran, the following are recommended

1. Reducing infertility treatment costs;
2. Improving economic conditions and reducing unemployment;
3. Strengthening job and financial security;
4. Social support for women to have children and creating suitable working conditions during pregnancy;
5. Reducing the distribution of contraceptives;
6. Culturization and educating people about the consequences of single-childhood;
7. Planning and educating toward facilitating marriage, lowering the age of marriage and strengthening the family;
8. Improving the physical and mental preparation of young people for raising the next generation and pregnancy;
9. Providing financial incentives and banking facilities to families with children.

## References

[1] Jafari H, Jaafaripooyan E, Vedadhir AA, Rahimi Foroushani A, Ahadinejad B, Pourreza A (2016). Socio-economic factors influencing on total fertility rate in Iran: a panel data analysis for the period of 2002-2012. Electron Physician.

[2] Pourreza A, Sadeghi A, Amini-Rarani M, Khodayari-Zarnaq R, Jafari H (2021). Contributing factors to the total fertility rate declining trend in the Middle East and North Africa: a systemic review. J Health Popul Nutr.

[3] McDonald P, Hosseini-Chavoshi M, Abbasi-Shavazi MJ, Rashidian A (2015). An assessment of recent Iranian fertility trends using parity progression ratios. Demogr Res.

[4] Wang H, Abbas KM, Abbasifard M, Abbasi-Kangevari M, Abbastabar H, Abd-Allah F (2020). Global age-sex-specific fertility, mortality, healthy life expectancy (HALE), and population estimates in 204 countries and territories, 1950-2019: a comprehensive demographic analysis for the Global Burden of Disease Study 2019. Lancet.

[5] Mohammad K, Khalaj Abadi Farahani F, Rahgozar M, Mahmoodi Farahani M (2002). Fertility in Islamic Republic of Iran: levels, trends and differentials during three decades (1967-1996). Can Stud Popul.

[6] Hosseini-Chavoshi M, Abbasi-Shavazi MJ, McDonald P (2016). Fertility, marriage, and family planning in Iran: implications for future policy. Popul Horizons.

[7] Mehri N, Messkoub M, Kunkel S (2020). Trends, determinants and the implications of population aging in Iran. Ageing Int.

[8] Hosseini-Chavoshi M, Abbasi-Shavazi MJ. Demographic transition in Iran: changes and challenges. In: Groth H, Sousa-Poza A, eds. Population Dynamics in Muslim Countries: Assembling the Jigsaw. Berlin, Heidelberg: Springer; 2012. p. 97-115. 10.1007/978-3-642-27881-5_7.

